# You have been terminated: robots, work, and taxation

**DOI:** 10.1007/s12232-023-00419-6

**Published:** 2023-05-17

**Authors:** Gizem Akar, Giorgia Casalone, Martin Zagler

**Affiliations:** 1 WU Vienna University of Economics and Business, Vienna, Austria; 2 UPO University of Eastern Piedmont, Novara, Italy

**Keywords:** Robots, Singularity, Robot taxation, Economic growth, O40, O33, H11

## Abstract

We present a three sector OLG model with a homogeneous output good that is produced with traditional or robot technology. The traditional sector produces with labor and capital, whereas the modern sector employs robots instead of labor. Robots and workers are modeled as perfect substitutes to investigate whether economic policy under the harshest assumptions is able to prevent the ascent of a robotized economy. While we find that the transition is inevitable, higher taxes on robots and revenues can slow down the process. We also that the economy will switch from an exogenous growth model based on TFP to an endogenous growth model due to constant returns with respect to reproducible factors of production as it becomes fully robotized.

## Motivation

The rise of robots is a popular theme in public debate. Already in 1952, in his novel Player Piano, Kurt Vonegut described a society where production is completely automated. Very few people work as engineers, the rest remain idle. In nonfiction literature, Yuval Noah Harari (2018) argues that the advent of robots and artificial intelligence will lead to mass unemployment. But the dystopian image most of us will have in mind when thinking about the impact of robots on the future of society is probably shaped by David Cameron’s film The Terminator (1984), starring Arnold Schwarzenegger (and inspiring the title of this paper).

Economists present a more balanced viewpoint. In his Essays in Persuasion (1930), John Maynard Keynes predicted that by 2030 there will be at most 15 h of work per week for each of us, the rest being replaced by machines. In a recent article, The Economist (11 Jan 2018) presents two differing opinions whether robots will eliminate jobs or not. The scientific discourse - to be reviewed in the next chapter - on the impact of robots on work is ongoing.

The ambition of this paper is to present an extreme case scenario where robots can indeed eliminate work. In such, it is a proof of concept of something that may happen, rather than a description of what will happen. The crucial assumption is that robots are modeled as perfect substitutes to physical labor. Under such conditions, we will demonstrate that work as we know it will end. We will also investigate whether (tax) policy has any possibilities at all to prevent such an event.

The paper proceeds as follows. The next chapter will review the literature on robots, with a special focus on the labor market, taxation and welfare. We will present the building blocks of the model in chapter 3, starting with households, and then discuss the three sectors of the economy, the traditional sector, which produces with capital and labor, the modern sector, which produces with capital and robots, and the robotics sector, which manufactures the robots. Chapter 4 then derives the equilibrium and the transition to a fully automated economy. We will discuss policy options in chapter 5, before summarizing and concluding.

## Related literature

### Robots and the labor market

Recent empirical and theoretical literature provides mixed results about the impact of robots on the labor market.

One of the earliest empirical investigations into the impact of robots on employment is Autor et al. (2003). They find that robots will replace jobs with a large content of routine tasks, whereas jobs with many non-routine tasks will actually benefit.

In their study, Acemoglu and Restrepo (2017), analyze the effect of the increase in industrial robot usage between 1990 and 2007 on USA local labor markets and estimate that one more robot per thousand workers reduces the employment to population ratio by about 0.18$-$0.34 percentage points and wages by 0.25$-$0.5. Frey and Osborne (2013) find that almost half of the total US population is at risk of being automated over the next two decades.

Graetz and Michaels (2018) use panel data on robot adoption within industries in 17 countries from 1993-2007, findings show that robots did not significantly reduce total employment even though they reduced low-skilled workers’ share.

David (2017) evaluates the risk of job destruction caused by computer technology in Japan. They find evidence that approximately 55% of jobs are susceptible to be carried by computer capital in the next years.

In contrast, Dixon and Hong (2020) find that robot adoption actually leads to an increase in employment. More importantly, they argue that robots will lead to an increase in high skilled and low skilled employment, whereas it will reduce employment of medium skilled managers. More importantly, they argue that robots will lead to organizational change, but this aspect is beyond the scope of this paper.

Dauth et al. (2017) look into the effects of industrial robots on the careers of individual manufacturing workers and the equilibrium impact on local labor markets in Germany. The important conclusion is that robots do not result in overall job losses unlike the situation in the US, but change the mixture of the aggregate employment in Germany. They estimate losses in the manufacturing sector but this was compensated with the additional jobs in the service sector.

Dengler and Matthes (2018) divide the tasks into groups (’routine versus non-routine’) to look into the substitution potentials of occupations for specifically Germany. When they use an ’occupation-level approach’, approximately 47% of the employees work in the substitutable occupations in 2013. Based on a ’task-based approach’, only 15% of workers are at risk of being replaced by automation. However, the authors emphasize that these are only the technical feasibilities. The link between automation probability and actual employment growth is not clear.

As another question, Zhang (2019) investigates whether the displacement of human workers by robots will widen the wage inequality between the skilled and unskilled labor and conclude that automation does not necessarily widen the wage gap. Guerreiro et al. (2017) show that without changes in the current US tax system, a sizable fall in the cost of automation would lead to a massive rise in income inequality.

The literature on the impact of robots on the labor market is predominantly empirical. This paper compliments the literature by providing a theoretical underpinning under extreme conditions, where labor is completely substitutable. This fits medium and (to a certain degree) low skilled work, and the model is consistent with the empirical evidence.

### Robotization and the welfare state

Potentially detrimental effects of automation lead policy-makers to think about several policy measures. These includes robot taxation, a universal basic income, higher education spending, and raising the marginal tax rates of high income individuals among others.

By using a DSGE model for the US economy, Peralta-Alva and Roitman (2018) look into the policies to adjust the economy to technology shocks (an automation shock and a drop in the price of capital). Changing the distribution of market income through education and other human capital formation policies or adjusting the incomes through tax cuts/benefits are some of the options depending on the society’s preference on equality or higher output. Based on US education data, financing higher education spending requires an increase of 2.5 percentage points in consumption taxes relative to a no-education-policy response baseline.

Goolsbee (2018) considers fiscal policy in an artificial intelligence intensive economy. In the case where nothing slows the speed of AI adoption and there is a mass job displacement in a short time, there is a call for a Universal Basic Income (UBI) introduction. Yet, there are number of challenges associated with negative taxes and UBI as a policy solution. It is likely to expect a sizable drop in labor market participation by low wage earners and worsen the non-participation rate in the economy.

Guerreiro et al. (2017) ask how should the government policy respond to technological change. They have a different attitude toward universal basic income. Their model demonstrates a massive rise in income inequality if automation costs fall. This can be reduced by making the tax system more progressive and by taxing robots, but this comes with a price; i.e., efficiency loss. This can be improved with Mirrlesian optimal income tax but as an alternative approach, when the transfer of basic income in place, it is optimal to tax robots as long as there is partial automation.

In their recent research report, Petropoulos et al. (2019) argue that digitization and automation will lead to a change from dependent employment to self employment, thus putting strains on the Bismarckian concept of the welfare state. They suggest to include self employed into the social protection system, but fall short of discussing potential issues with structurally unemployed.

This paper, too, is concerned with policy. If demand for labor vanishes, clearly taxation and redistribution is the only solution to maintain the welfare of the population. This paper enables use to evaluate welfare implications of such a government policy reform. It is thus close to Guerreiro et al. (2017), but as labor demand is eliminated in the long run in our model, progressive taxation would not suffice.

### Robot taxation

Robot tax literature investigates whether it is optimal to tax robots and if yes, what would be the efficient tax rate. There are different approaches and conclusions to robot taxation. Gasteiger and Prettner (2017) analyze the long-run growth effects of automation in the canonical overlapping generations model framework and conclude that automation does not lead to positive long-run growth. On the production side, they introduce a robot tax to automation capital and show that in the steady state, it could raise the capital stock. Another paper from Zhang (2019) by using canonical specific-factor framework, concludes that a tax on robots does always improve wage inequality. Guerreiro et al. (2017) states that it is optimal to tax robots as long as lump-sum transfers and partial automation in place.

Costinot and Werning (2018) explore the magnitude of optimal taxes on robots and trade. They find the efficient tax rate on robots ranges from 1% to 3.7%. They then ask whether robots should be taxed more as they get cheaper and cheaper with the improvements in automation. Despite a strict preference for redistribution by government and increasing inequality because of automation, the authors show that new technologies are associated with lower taxes on firms using those technologies. Their result is that even if automation distorts wages of low skilled workers and redistribution is important for the economy, this does not justify the rationale for taxes and subsidies on innovation to distort technology adoption by firms.

Thuemmel (2022) studies the optimal taxation of robots and labor income. The author shows that it is optimal to distort robot adoption when measured against (a far more distortionary) income tax on labor. The optimal tax for the US is positive once robots are sufficiently cheap and generates small welfare gains.

This paper, too, theoretically investigates the impact of taxation on robotization. Within a positive analysis, we can confirm that robot taxation reduces the speed of adoption as in the previous literature. The novel contribution here is that from a welfare perspective, we find that a tax on robots reduces sunk fixed costs and therefore is welfare improving.

## The model

### Households

#### A general framework

Consider an overlapping generations model where households live for two periods. They supply labor when they are young and live from savings when they are old. We normalize the number of households to unity. Households maximize a generic utility function,

1 $$\begin{aligned} \mathrm{{max}} \, U(C_t, C_{t+1}, L_t). \end{aligned}$$

In the first period of their lives, households consume, suffer from work (disutility of labor) and save all their wage income and possible government transfers net of first period consumption,

2 $$\begin{aligned} S_t = w_t L_t - C_t + \tau ^y_t. \end{aligned}$$

In old age, households consume all their savings and possible government transfers,

3 $$\begin{aligned} C_{t+1} = (1 + r_t) S_t + \tau ^o_{t+1}. \end{aligned}$$

It is rather straightforward to collapse the last two equations into a single intertemporal budget constraint,

4 $$\begin{aligned} C_{t+1} = (1 + r_t) (w_t L_t - C_t + \tau ^y_t) + \tau ^o_{t+1}. \end{aligned}$$

Note that we would have a public pension system with $\tau ^y_t = 0$ and $\tau ^o_t > 0$ and a universal basic income with $\tau ^y_t = \tau ^o_t > 0$. None of the benefits can be linked to choices by individual households and would have to be financed out of taxes on firms and factors of production. We would have a negative income tax with $\tau ^y_t = \tau w_t L_t$. We would have unemployment benefits if $\tau ^y_t = \mathrm{{max}}(b_t, w_t L_t)$.

Substituting the budget constraint (4) into the utility function (1) and taking derivatives with respect to first period consumption yields the following first order condition for household utility maximization,

5 $$\begin{aligned} U_1(C_t, C_{t+1}, L_t) - (1 + r_t) U_2(C_t, C_{t+1}, L_t) = 0, \end{aligned}$$

where $U_1$ is the first derivative of the utility function with respect to its first element $(C_t)$ and $U_2$ with respect to the second element $(C_{t+1})$. Rearranging this first order condition gives a conventional intertemporal Euler equation,

6 $$\begin{aligned} \frac{U_1}{U_2} = (1 + r_t). \end{aligned}$$

Shifting consumption from present to the future will lead to an increase in the marginal utility from first period consumption $U_1$ and to a decline in the marginal utility of second period consumption $U_2$. Households will shift consumption to the future until the marginal rate of substitution equals the relative price, in this cast the interest factor $1 + r_t$.

Taking derivatives with respect to labor yields the first order condition for household utility maximization,

7 $$\begin{aligned} (1 + r_t) w_t U_2(C_t, C_{t+1}, L_t) + U_3(C_t, C_{t+1}, L_t) = 0. \end{aligned}$$

Eliminating marginal utility of second period consumption with the intertemporal Euler Eq. (6) gives

8 $$\begin{aligned} \frac{U_3}{U_1} = w_t. \end{aligned}$$

The marginal rate of substitution between labor and leisure equals the opportunity cost of work $w_t$. The two first order conditions, Eqs. (6) and (8) together with the budget constraint (4) implicitly identify the choice of households over consumption and leisure.

#### A special case

Whilst it is tempting to continue with the general framework, it would refrain us from obtaining a closed form solution, so we will discuss a special case next. Note however that all qualitative results would carry over in the general case. We will assume that the utility function is additively separable and that utility is linear in consumption,

9 $$\begin{aligned} U(L_t, C_{t+1}) = \beta C_t + C_{t+1} - u(L_t), \end{aligned}$$

where $\beta$ determines the preference for present consumption over future consumption, or the degree of impatience. The assumption of additive separability implies that the level of consumption has no impact on the trade-off between work and leisure. The assumption that consumption enters linear implies that there is no diminishing marginal product of consumption, which is a strong assumption, but consistent with the Kuznet’s consumption function adopted in Solow (1956). Both assumptions are qualitatively unproblematic.

The marginal rate of substitution between current and future consumption for the above utility function (9) equals

10 $$\begin{aligned} \frac{U_1}{U_2} = \beta . \end{aligned}$$

Compared with the first order condition (5), we immediately realize that we are in a corner solution. In case $\beta > 1 + r_t$, all consumption would happen in the first period, and there would be no capital accumulation. We would thus be stuck in a forager society forever. The more interesting case, and the one we will pursue, is $\beta < 1 + r_t$, and all consumption will be deferred to the second period.

Disutility from work equals $u(L_t)$ with $u'(L_t)>0$ and $u"(L_t)>0$. This assumption ensures that an increase in work reduces utility (the marginal utility of labor is negative), and that an increase in wages will lead to an increase in labor supply. This will simplify the first order condition with respect to labor to

11 $$\begin{aligned} (1 + r_t) w_t + u'(L_t) = 0. \end{aligned}$$

Multiplying Eq. (11) with $L_t$ and substituting into the budget constraint (4) yields,

12 $$\begin{aligned} C_{t+1} = u'(L_t) L_t + (1 + r_t) \tau ^y_t + \tau ^o_{t+1}. \end{aligned}$$

Note that it makes little sense for a government to pay transfers to households who do not have (consumption) expenditures yet, so we will set $\tau ^y_t = 0$ in most cases.

### The manufacturing sectors

There will be a single homogeneous output good in the economy. It can be produced either with a traditional technology that uses capital and labor as inputs, or with a modern technology that uses capital and robots as inputs. Both the traditional and modern sectors operate under constant returns to scale with an identical output elasticity of capital, $\alpha$. This implies identical output elasticities for labor and robots, and therefore that labor and robots are prefect substitutes. This is a crucial assumption for the model. It presents the worst case scenario for employment, with the ambition to see whether there is a future for work under extreme conditions. We may still imagine that consumers have preferences for human made products.^1^ We could model this with a consumption bundle that consists of both human made and machine products with a finite (constant) elasticity of substitution (CES). This would lead to some human work remaining forever. However, this impedes us from finding a closed form solution, so for the sake of simplicity we refrain from this assumption.

Firms hire workers from the household sector and rent capital and robots from the old. Given that this is a two period OLG model, assuming full depreciation of the capital and robots good seems reasonable. Production is given by

13 $$\begin{aligned} Y^S_t = (K^S_t)^{\alpha } (A_t X_t)^{1-\alpha }. \end{aligned}$$

where $S \in \{T, M\}$ indicates the traditional (*T*) and modern (*M*) sector, respectively. The second production factor $X_t \in \{L_t, R_t\}$ is either labor $L_t$ (in the traditional sector) or robots $R_t$ (in the modern sector). We assume that labor augmenting technical progress $A_t$ grows at a constant rate $g_A$. Normalizing the price of the output good to unity, profits are given by

14 $$\begin{aligned} \pi ^S_t = (1 - \tau ^S) (K^S_t)^{\alpha } (A_t X_t)^{1-\alpha } - (1 + \tau ^X) q_t X_t - (1 + \tau ^K) (1 + r_t) K^S_t, \end{aligned}$$

where we have introduced a tax on turnover (VAT), $\tau ^S$, a tax on capital inputs (capital income tax), $\tau ^K$ , and a tax on the other factor, $\tau ^X$, which can be either a personal income tax in the case of labor, or a robot tax. As capital fully depreciates within one period, firms are expected to pay for the full capital good and interest, hence the net user cost of capital equals the interest factor $1 + r_t$. The rental price for the other factor $X_t$ equals $q_t \in \{w_t, p_t\}$. The first order condition with respect to capital reads

15 $$\begin{aligned} \frac{d\pi ^S_t}{dK^S_t} = \alpha (1 - \tau ^S) (K^S_t)^{\alpha -1} (A_t X_t)^{1-\alpha } - (1 + \tau ^K) (1 + r_t), \end{aligned}$$

which should equal zero in optimum and can be simplified to

16 $$\begin{aligned} \alpha (1 - \tau ^S) Y^S_t = (1 + \tau ^K)(1 + r_t) K^S_t. \end{aligned}$$

In theory we could distinguish between a tax on capital in the traditional versus the modern sector, but this is equivalent to a tax on output of the two sectors, as shown in Eq. (16) above. The first order condition with respect to the other factor yields

17 $$\begin{aligned} \frac{d\pi ^S_t}{dX_t} = (1 - \alpha ) (1 - \tau ^S) (K^S_t)^{\alpha } A_t^{1-\alpha } X_t^{-\alpha } - (1 + \tau ^X)q_t, \end{aligned}$$

which should equal zero in optimum and can be simplified in the case of labor in the traditional sector to

18 $$\begin{aligned} (1 - \alpha ) (1 - \tau ^T) Y^T_t = (1 + \tau ^W)w_t L_t. \end{aligned}$$

Substituting (16) and (18) into (14) shows that firms in the traditional sector make zero profits. With constant returns to scale according to (13), the size of the firm is therefore undetermined, and can be either infinitely small or large. For the sake of simplicity, we assume that there is a single firm in the traditional sector operating under perfect competition. Substituting (16) and (18) into (13) allows us to determine relative prices,

19 $$\begin{aligned} w_t^{1-\alpha } (1 + r_t)^{\alpha } = \alpha ^{\alpha } (1 - \alpha )^{1-\alpha } \frac{1 - \tau ^T}{1 + \tau ^W} \left( \frac{1 + \tau ^W}{1 + \tau ^K} \right) ^{\alpha } A_t^{1-\alpha }. \end{aligned}$$

Simplifying the first order condition (17) in the modern sector yields,

20 $$\begin{aligned} (1 - \alpha ) (1 - \tau ^M) Y^M_t = (1 + \tau ^R) p_t R_t. \end{aligned}$$

Solving for the price of robots $p_t$, this equation gives a demand function for robots. Substituting (16) and (20) into (14) shows that firms in the modern sector make zero profits. With constant returns to scale according to (13), the size of the firm is therefore indetermined. Without loss of generality, we can assume that there is a single firm in the modern sector operating under perfect competition, too.

### The robotics sector

Robots are not standard manufacturing products and certainly require an enormous amount of knowledge and R &D to produce. We therefore assume that there are only a few firms (*n*) supplying robots, who work under imperfect competition (Cournot oligopolists).

Each robotics firm *i* uses the homogeneous output good ($Z_{i,t}$) produced by the manufacturing sectors to transform it into robots $R_{i,t}$ given productivity $B_t$,

21 $$\begin{aligned} R_{i,t} = B_{t+1} Z_{i,t}, \end{aligned}$$

where $R_{t} = \sum {R_{i,t}}$ and $Z_t = \sum {Z_{i,t}}$. Profits of a particular robotics firm $\pi _{i,t}^R$ therefore depend on revenues from sales minus costs. Firms can enter freely into the production of robots as long as they pay a fixed cost for using a robot blueprint $F_{i,t}$,

22 $$\begin{aligned} \pi _{i,t}^R = p_{i,t} R_{i,t} - Z_{i,t} - F_{i,t}. \end{aligned}$$

All robotics firms provide identical products for the same market with identical technology, so by symmetry all robotics firms will set the same price $p_{i,t} = p_t$. Substituting technology (21) and the demand function (20) gives

23 $$\begin{aligned} \pi _{i,t}^R = (1 - \alpha ) \frac{1 - \tau ^M}{1 + \tau ^R} \frac{Y^M_t}{R_t} R_{i,t} - \frac{R_{i,t}}{B_t} - F_{i,t}. \end{aligned}$$

Note that the oligopolistic firm knows that its supply of robots $R_{i,t}$ has an impact on total supply of robots $R_t$ and production in the modern sector $Y^M_t$, and therefore of the price for the robots $p_t$, hence

24 $$\begin{aligned} \frac{\mathrm{{d}}\pi _{i,t}^R}{\mathrm{{d}}R_{i,t}} = (1 - \alpha ) \frac{1 - \tau ^M}{1 + \tau ^R} \frac{Y^M_t}{R_t} \left( 1 + (1 - \alpha ) \frac{R_{i,t}}{R_t} - \frac{R_{i,t}}{R_t}\right) - \frac{1}{B_t}. \end{aligned}$$

With *n* symmetric firms, we will have $R_t / R_{i,t} = n$. In optimum, $\mathrm{{d}} \pi _{i,t} / \mathrm{{d}}R_{i,t} = 0$, so that we obtain robot supply,

25 $$\begin{aligned} R_t = \eta \frac{1 - \tau ^M}{1 + \tau ^R} B_t Y^M_t \end{aligned}$$

where $\eta = (1 - \alpha )( 1 - \alpha / n)$. In the case of a monopolist supplier, this would reduce to $(1 - \alpha )^2$, which is the well-known result of double marginalization. Free entry implies that firms will enter the robotics industry until profits (23) are zero. Substituting (25) into (23) and assuming that aggregate fixed costs are proportional to output, $nF_{i,t} = f Y^M_t$ yields after rearrangement the number of firms producing robots,

26 $$\begin{aligned} n = \frac{\alpha (1 - \alpha )}{f} \frac{1 - \tau ^M}{1 + \tau ^R} \end{aligned}$$

We can then derive the Amoroso-Robinson rule for mark-up pricing by substituting (25) into demand for robots (20),

27 $$\begin{aligned} p_t = \frac{n}{n - \alpha } B^{-1}_t. \end{aligned}$$

Productivity in robotics will grow at $g_B$, thus reducing costs of robots (and hence their price) over time. An increase in the number of firms will lower the price for robots. According to (26), the number of firms will increase with a fall in fixed costs *f* and a fall in the taxes on the modern sector $\tau ^M$ and robots $\tau ^R$. As the number of robotics firms goes to infinity and market power vanishes, the price of robots will equal the cost of robots, $1/B_t$.

## Equilibrium

The traditional and the modern sector both produce the same output good, which can be used for consumption, capital and robot investment, so that market clearing reads,

28 $$\begin{aligned} Y^T_t + Y^M_t = C_t + K^T_{t+1} + K^M_{t+1} + Z_t. \end{aligned}$$

### A purely traditional economy

In the absence of a robotics sector, $Y^T_t = K^M_t = Z_t = 0$ for all *t*, and the model simplifies to a very conventional Solow OLG model. Market clearing (28) will reduce to $K^T_{t+1} = Y^T_t - C_t$. Substituting traditional technology (13) and the household optimum yields,

29 $$\begin{aligned} K^T_{t+1} = (K^T_t)^{\alpha } (A_t L_t)^{1-\alpha } - u'(L_{t-1}) L_{t-1} - (1 + r_{t-1}) \tau ^y_{t-1} - \tau ^o_{t}. \end{aligned}$$

Setting first period transfers to zero, $\tau ^y_{t-1} = 0$, this defines a dynamic equation in the capital stock. Dividing both sides by $K^T_t$ gives the growth factor of the purely traditional economy,

30 $$\begin{aligned} 1 + g_K = \left( \frac{K^T_t}{A_t L_t} \right) ^{\alpha -1} - \frac{ u'(L_{t-1}) L_{t-1} - (1 + r_{t-1}) \tau ^y_{t-1} - \tau ^o_{t}}{K^T_t}, \end{aligned}$$

As the capital stock in the economy grows, the second term converges to zero. In this case, however, pension payments will become more and more negligent with respect to income, so we may want to assume transfers proportional to income, $\tau _t^o = \tau Y_t$. In this case the long run equilibrium growth rate of the capital stock (29) will change to

31 $$\begin{aligned} 1 + g_K = (1 - \tau ) \left( \frac{K^T_t}{A_t L_t} \right) ^{\alpha -1}. \end{aligned}$$

Subsitutiting tradtitional technology (13) and the first order condition with respect to capital (16) gives

32 $$\begin{aligned} 1 + r^T = \frac{\alpha }{1 - \tau } \frac{1 - \tau ^T}{1 + \tau ^K}. \end{aligned}$$

This ensures that the interest rate is constant along the balanced growth path. Expectedly, a higher tax on capital and output of the traditional sector both reduce the interest rate. Note that an increase in pensions $\tau$ also reduces the interest rate, thus making work less attractive for private savings.

Along a balanced growth path we must have $g_K = g_A + g_L$, and substituting this into the production function, we also obtain $g_T = g_K$. From the first order condition of households (7) we obtain $g_w = -\epsilon g_L$, where $\epsilon$ is the labor supply elasticity $\epsilon = u"(L_t) L_t / u'(L_t)$. Substituting this into the first order condition for labor (18) yields $g_T = (1-\epsilon ) g_L$. Substituting this into the balanced growth rate of output, we find $g_T = [(\epsilon - 1)/\epsilon ] g_A$.

A purely traditional economy is following a conventional Solow growth model. In the long run, economic growth is driven by productivity and population growth. The size of the economy depends on deep parameters of the model $(\alpha )$ and tax rates. An increase in the tax on output $(\tau ^T)$ and capital $(\tau ^T)$ will both lower the interest rate and hence the capital stock in the steady state, which has a negative impact on the size of the economy.

### A purely robotized economy

In an economy that uses only the modern technology, labor demand falls to zero, implying $w_t = 0$. Hence, consumption will depend solely on government transfers, $C_t = \tau ^o_t$. The market clearing condition (28) therefore reduces to

33 $$\begin{aligned} K^M_{t+1} + R_t = Y^M_t - \tau ^o_t. \end{aligned}$$

Eliminating $Y^M_t$ by substituting (16) into (25) gives a constant proportion of robots and capital in production,

34 $$\begin{aligned} R_t = \left[ (1 - \alpha ) \frac{1 - \tau ^M}{1 + \tau ^R} - f\right] B_t Y^M_t, \end{aligned}$$

Substituting this into market clearing (33) and once again assuming that transfers are proportional to income $\tau ^o_t = \tau Y^M_t$, we obtain a dynamic equation in the capital stock,

35 $$\begin{aligned} K^M_{t+1} = \left[ 1 - \tau - (1 - \alpha ) \frac{1 - \tau ^M}{1 + \tau ^R} B_t + fB_t\right] \left[ (1 - \alpha ) \frac{1 - \tau ^M}{1 + \tau ^R} - f\right] ^{\frac{1 - \alpha }{\alpha }} (A_t B_t)^{\frac{1 - \alpha }{\alpha }} K^M_t \end{aligned}$$

which identifies the growth factor of the capital stock $K^M_{t+1} / K^M_t = 1 + g_K$. From (34), we know that this is identical to the growth factor in robots, and from (13) to output, $g_K = g_R = g_M$. Even in the absence of technical progress $A_t = B_t = 1$, the economy will exhibit long-run endogenous growth due to constant returns to scale with respect to reproducible factors of production, $g_M > 0$.

A purely robotized economy exhibits constant returns with respect to all reproducible factors of production, capital $K_t$ and robots $R_t$ and thus resembles an endogenous growth model. The growth rate of the economy itself depends on tax rates. An increase in the tax rate on output $(\tau ^M)$ and the tax rate on capital $(\tau ^R)$ will lead to an equivalent decline in economic growth. In the next chapter, we will analyze how the economy shifts from a traditional to a modern technology. Along this structural change, we will also find that we will switch from an exogenous growth model as identified in the previous chapter to the endogenous growth model of this chapter.

### The mixed economy

Substituting the modern technology expansion path (34) into technology (13) gives

36 $$\begin{aligned} Y^M_t = K^M_t \left[ (1 - \alpha ) \frac{1 - \tau ^M}{1 + \tau ^R} - f\right] ^{\frac{1 - \alpha }{\alpha }} (A_t B_t)^{\frac{1 - \alpha }{\alpha }}. \end{aligned}$$

We can then derive the interest factor from the first order condition for capital in the modern economy (16),

37 $$\begin{aligned} 1 + r_t^M = \frac{1}{\alpha } \frac{1 - \tau ^M}{1 + \tau ^K} \left[ (1 - \alpha ) \frac{1 - \tau ^M}{1 + \tau ^R} - f\right] ^{\frac{1 - \alpha }{\alpha }} (A_t B_t)^{\frac{1 - \alpha }{\alpha }}. \end{aligned}$$

This is the demand function for capital goods in the modern sector, and it turns out to be perfectly elastic. If interest rates in the traditional sector are higher than the above, no capital will be invested in the modern sector, and the economy will be purely traditional. As interest rates in the modern sector increase, they will slowly siphon capital away from traditional firms, increasing their marginal product of capital (16), until at one point the traditional sector disappears.

#### The rise of the machines

In the absence of a disutility of work, and if the traditional sector is in steady-state, we can identify the advent of the singularity,^2^ when a fully robotized sector starts to emerge $(r^M \ge r^T)$,

38 $$\begin{aligned} A_t B_t \ge \left( \frac{\alpha ^2}{1 - \tau } \right) ^{\frac{\alpha }{1 - \alpha }} \left( \frac{1 - \tau ^T}{1 - \tau ^M} \right) ^{\frac{\alpha }{1 - \alpha }} \left[ (1 - \alpha ) \frac{1 - \tau ^M}{1 + \tau ^R} - f\right] ^{-1} \end{aligned}$$

Tax policy can delay the rise of robots. Higher taxes on revenues of the modern sector with respect to the traditional sector would obviously yield this result. However, this may be very hard to reach practically. Interestingly, a higher tax on robots $(\tau ^R)$ and a higher general tax level $(\tau ^M)$ would also retard the rise of robots. By contrast, productivity gains, both in the general economy $(A_t)$ and in the robotics sector $(B_t)$ will make fully automated production more likely.

#### Hasta la vista

By substituting interest rates in the mixed economy (37) into the equation determining relative prices (19), we obtain the evolution of wages in the mixed economy,

39 $$\begin{aligned} w_t = \alpha ^{\frac{2 \alpha }{1 - \alpha }} \frac{1 - \alpha }{1 + \tau ^W} \left( \frac{1 - \tau ^T}{1 - \tau ^M} \right) ^{\frac{\alpha }{1 - \alpha }} \left[ (1 - \alpha ) \frac{1 - \tau ^M}{1 + \tau ^R} - f\right] ^{-1} B^{\alpha - 1}. \end{aligned}$$

Clearly, an increase in the tax on labor ($\tau ^W$) will reduce (net) wages. The possibility to tax revenues of the traditional sector ($\tau ^T$) less than the modern sector would ($\tau ^M$) would improve wages, as the modern sector would take longer to evolve. Taxing robots would reduce entry in the robotics industry, render robots more expensive, and delay the decline in wages. Finally, we find that an increase in the productivity of the robotics sector (*B*) would lead to a decline in wages.

Having identified interest rates (37) and wages (39) of the mixed economy, we can implicitly derive employment from the household first order condition (11),

40 $$\begin{aligned} u'(L_t) = (\alpha - 1) \alpha ^{\frac{\alpha }{1 - \alpha }} \frac{1 + \tau ^W}{1 + \tau ^K} \left( \frac{1 - \tau ^T}{1 - \tau ^M} \right) ^{\frac{\alpha }{1 - \alpha }} \left[ (1 - \alpha ) \frac{1 - \tau ^M}{1 + \tau ^R} - f\right] ^{\frac{1}{\alpha }} A_t^{\frac{1 - \alpha }{\alpha }} B^{\frac{(1 - \alpha )^2}{\alpha }}. \end{aligned}$$

Note that the right hand side of the above equation is negative, as $\alpha - 1 < 0$. This implies that productivity gains both in production (*A*) and robotics (*B*) reduce employment. Three tax wedges are important to understand the evolution of employment. Revenue taxation between the traditional and modern sector has no impact on employment unless differentiated. Taxes on labor will reduce employment, unless capital - the substitute of labor in the traditional sector - is equally taxed. Finally, robot taxation reduces incentives to invest in robots and this slows the process of transformation of the economy.

## Taxation and robotization

In order to be able to tax robots, we need to be able to define them. The International Organisation for Standardisation (2012) defines an industrial robot as “an automatically controlled, reprogrammable, multipurpose manipulator programmable in three or more axes, which can be either fixed in place or mobile for use in industrial automation applications”. This definition has also been taken up by the International Federation of Robotics. Kaplan (2015) defines robotic systems as “sensors and actuators that can see, hear, feel (touch), smell, [taste] and interact with their surroundings”. The EU Parliament (2017) defines smart robots as “the acquisition of autonomy through sensors and/or by exchanging data with its environment (interconnectivity) and the trading and analysis of those data; self-learning from experience and by interaction (optional criterion); at least a minor physical support; the adaptation of its behavior and actions to the environment; and the absence of life in the biological sense”.

All three definitions give the reader a clear idea what constitutes a robot. However, they are of little practical use when it comes to taxation, as an inverted Turing test, that we have devised can show: Under all of the above definitions, a modern car could be classified as a robot, whereas your typical Star Wars Fifth Class Service Droid (GNK Series) would be able to escape the definition. If robots are programmed to tell the truth, self-declaration would work. But robots programmed to maximize profits would certainly self-declare as a simple machine if robots are taxed higher than machines. For all practical purposes, we will therefore assume that a robot cannot be differentiated from capital, and hence we will assume $\tau ^R = \tau ^K$ and $\tau ^T = \tau ^M$.

### Proposition 1

With exogenous technical progress, the rise of the modern sector cannot be blocked. It can be slowed down by an increase in fixed costs to enter the robotics sector (*f*), an increase in the tax on machines $(\tau ^R)$, revenues $(\tau ^M)$, and transfers $(\tau )$.

An increase in the fixed costs in the robotics industry providers will increase the mark-up for robots, and this makes modern technology more costly, and traditional technology can prevail for longer. Similarly, taxes on robots make entry less profitable and thus slow the rise of the modern sector. An increase in proportional transfers introduces a tax wedge in capital accumulation, leading to an increase in the interest rate of the traditional sector, and thus rendering investment in traditional technology profitable for longer. These results can be immediately observed from equation (38).

Instead of preventing the emergence of a modern sector, politics may simply aim at reducing the use of robots within modern technology. With the exemption of reducing competition in the robotics sector, and a legal ban, there is little that can be achieved through taxation, as can be observed from Eq. (34). The same holds for ambitions to stop the decline of the traditional sector, Eq. (36).

### Proposition 2

Lower taxes on wages and output (of the traditional sector) will foster wages, just like a increase in costs or a tax-induced decline of revenues of the robotics sector.

An reduction in the tax on labor will obviously increase the after-tax real wage. Interestingly, a reduction in the tax on output of the traditional sector will increase in the amount of income distributed to workers and thus improve wages, despite the fact that this will also happen in the modern sector contemporaneously, given $\tau ^T = \tau ^M$. As we can observe from Eq. (39), a slower rise of the robotics sector (identified by the square brackets) will also raise wages.

The real issue is of concern over the impact of robots on employment. Here, our results depend crucially on the supply of labor.

### Proposition 3

There is no revenue neutral tax reform that can stop the decline of employment in a robotized economy. Tax policy can however slow down the transition of the economy.

With technological progress in production, (*A*), and robotics, (*B*) ever increasing, the inequality (38) will eventually be satisfied, irrespective of the implemented tax rates. However a tax reform that burdens labor less at the expense of higher taxes on capital, $(\tau ^K)$, and robots, $(\tau ^R)$, will lead to higher employment for every level of technological progress, (*A*) and (*B*).

## Welfare

There are no pure profits in this model, so welfare of the representative household depends on labor income and transfers. In a purely traditional economy consumers receive wages $w_t$ when young, transfers $\tau _{t+1}^o$ when old and face disutility from work. Given that utility functions are unique up to a linear transformation, we can say nothing about the level^3^ of the felicity function of leisure, $u(L_t)$. However, as the economy grows and grows, this term becomes less and less important, so that we can ignore it in the limit. Thus, utility (9) of the representative household in a traditional economy can be approximated by

41 $$\begin{aligned} u_t^T = (1 - \alpha + \tau ) Y^T, \end{aligned}$$

where we have made use of the fact that the labor share in income is $(1 - \alpha$). By contrast, in a purely robotized economy, there is no room for labor income and the entire household income depends on transfers. In order to compensate households for the loss of labor income government would need to set a much higher transfer rate $(\tilde{\tau })$ in the case of a fully robotized economy,

42 $$\begin{aligned} \tilde{\tau } = \tau + 1 - \alpha . \end{aligned}$$

Obviously, given a balanced budget, higher transfers require higher taxes on output $(\tau ^M)$ or capital in the modern economy $(\tau ^R)$. As proposition (1) has shown, this will not stop the modern economy from emerging, but at best slow down its advent. As shown in chapter 4.3, the modern economy will only come about if output exceeds the traditional economy, $Y^M > Y^T$, hence a sufficient condition that the modern economy is welfare increasing^4^ is for transfers to equal or exceed $\tilde{\tau }$.

Whilst taxes on output $(\tau ^M)$ and robots $(\tau ^M)$ have an identical impact on economic growth, Eq. (35), there may be a less distortionary way to finance transfers to households. As every additional firm in the oligopolistic robotics sector requires fixed costs $f_i$, a tax on profits of the robotics firms would increase efficiency of the sector and thus lead to an increase in output $Y^M$.

## Conclusions

We have presented a three sector overlapping generations model with a homogeneous output good that is produced with traditional or modern technology. The traditional sector produces with labor and capital, whereas the modern sector employs robots instead of labor. Labor and robots are thus perfect substitutes. This is the crucial assumption of the paper. We have investigated - as a proof of concept - if robots will be able to take over jobs from people. The robotics sector produces robots using the homogeneous output good. In this setting, we were able to obtain several novel results.

First, unless exogenous technical progress comes to a complete halt, the economy will shift from a production model based on capital and labor to a model based on physical capital and robots. The paper identifies structural change in the economy. Interestingly, with decreasing returns to scale with respect to reproducible factors of production, an economy based on a traditional sector follows an exogenous growth model. However, as both physical capital and robots are reproducible, the economy will transition to an endogenous growth model as the economy substitutes all labor with robots.

This transition to a modern economy based on capital and labor cannot be stopped. We find that wages fall with a relative increase in productivity and robots will more and more replace workers in the production process. Employment will decline along with the rise of robots.

Whilst tax policy cannot (and from a welfare perspective should not) prevent a modern economy from emerging, it can slow the speed of transition. It may be tempting to tax revenues of the modern sector or robot inputs in production. But this may be practically impossible, as it may be difficult to distinguish robots from physical capital and the homogeneous output good produced with traditional technology from one produced with modern technology. However, we have demonstrated higher tax rates can reduce the speed of transition. First, a tax on machines (capital and robots) will increase costs for robots with respect to labor, and slow down their adaptation. Second, a tax on revenues (both in the traditional and the modern sector) will lead to lower profits from the sale of robots, and this will also somewhat halt the transition to a robotized economy. Finally, higher transfers to households will increase interest rates and thus returns of the traditional sector, greasing the wheels of transition. As these transfers need to be financed somehow, this also points toward higher taxes. In summary, we find that economies with higher taxation may transition to a fully robotized economy lateron. But the rise of robots is inevitable.

## Footnotes

1 Medical services would be a good example.

2 The singularity actually occurs not when fully automated production starts, but when computers become self aware. We apologize for this imprecision here. As fans of the Terminator movie know, it took Skynet a mere two hours and 14 min to become self-aware on Judgement Day, August 4, 1997.

3 All that is required to justify positive wages is a marginal disutility of work, $du(L_T)/dL_t > 0$.

4 We can think of this as the *Star Trek next Generation* version of the future, where all material needs have been taken care of by technology. As a peculiarity, note that in the absence of transfers, $\tilde{\tau } = 0$, all output would be reinvested either as capital or robots (and investment would become a sink), not unlike the dystopian view of *Terminator*.
